# Ulceration of the Tonsils

**Published:** 1868

**Authors:** 


### ULCERATION OF LHE TONSILS.

Dr. G. W. Champ recommends the following wash as most effectual in ulceration of the tonsils, or apthous affections of the mouth: Take pulv. sulphate of zinc and chlorate of potash, of each two drachms; strong sage tea, half a pint. Mix. Gargle the throat frequently.—*Ibid.*

